# Annual acknowledgement of manuscript reviewers

**DOI:** 10.1186/s13584-015-0001-3

**Published:** 2015-02-03

**Authors:** Bruce Rosen, Avi Israeli

**Affiliations:** Israel Journal of Health Policy Research (IJHPR), London, UK

## Abstract

The Editors of *Israel Journal of Health Policy Research* would like to thank all reviewers, both external and Editorial Board Members, who have contributed to the journal in 2014, and whose valuable support continues to be essential to the success of the journal.

**David Abrams**

United States of America

**Linda H Aiken**

United States of America

**Moty Amar**

Israel

**Gilhar Amos**

Israel

**Nachman Ash**

Israel

**Igor Barash**

Israel

**Orna Baron-Epel**

Israel

**Ilana Belmaker**

Israel

**Netta Bentur**

Israel

**Bobbie Berkowitz**

United States of America

**Gabi Bin Nun**

Israel

**Sue Bogner**

United States of America

**Malke Borow**

Israel

**Shuli Brammli-Greenberg**

Israel

**Mayer Brezis**

Israel

**Timothy Brown**

United States of America

**Yeezkel Caine**

Israel

**Ronit Calderon-Margalit**

Israel

**Michael Chaiton**

Canada

**David Chinitz**

Israel

**Gabriel Chodick**

Israel

**Jeannie Cimiotti**

United States of America

**Daniel Cohen**

Israel

**Marc Cohen**

United Arab Emirates

**Robert Cohen**

Jordan

**Nihaya Daoud**

Israel

**Aad de Roo**

Netherlands

**Gordon DeFriese**

United States of America

**Marion Devaux**

France

**Francois Dionne**

Canada

**Milka Donchin**

Israel

**Avi Dor**

United States of America

**Israel Doron**

Israel

**Erin DuPree**

United States of America

**Eldad Elnekave**

Israel

**Ruth Endacott**

United Kingdom

**Ronit Endevelt**

Israel

**Paula Feder-Bubis**

Israel

**Roger Feldman**

United States of America

**Aharon Finestone**

Israel

**Shira Fischer**

United States of America

**Saralee Glasser**

Israel

**Shimon Glick**

Israel

**Michael Gordon**

Canada

**Dan Greenberg**

Israel

**Alexander Grinshpoon**

Israel

**Raz Gross**

Israel

**Itamar Grotto**

Israel

**Dror Guberman**

Israel

**Ziona Haklai**

Israel

**Jonathan Halevy**

Israel

**Pinchas Halpern**

Israel

**Paul Hansen**

New Zealand

**Naomi Hausman**

Israel

**David Hepner**

United States of America

**Andreas Hoeft**

Germany

**Tuvia Horev**

Israel

**Jonathan Huppert**

Israel

**Dena Jaffe**

Israel

**Marcia Javitt**

United States of America

**Jonathan Javitt**

Israel

**Patrick Jeurissen**

Netherlands

**Nir Kaidar**

Israel

**Orit Karnieli-Miller**

Israel

**Vladimir Khanassov**

Canada

**Shmuel Klang**

Israel

**Eileen Lake**

United States of America

**Bruce Landon**

United States of America

**Luís Lapão**

Portugal

**Leonard Leibovici**

Israel

**Gila Leiter**

United States of America

**Moshe Leshno**

Israel

**Hagai Levine**

Israel

**Osnat Levtzion-Korach**

Israel

**Noah Lewin-Epstein**

Israel

**Robert Like**

United States of America

**Orly Manor**

Israel

**Martin McKee**

United Kingdom

**Jacob Menczel**

Israel

**Tal Morginstin**

Israel

**Shlomo Mor-Yosef**

Israel

**Guy Mundlak**

Israel

**Yaron Mushkat**

Israel

**Leanne Nanttais-Smith**

United States of America

**Yehuda Neumark**

Israel

**Liebermann Nicky**

Israel

**Nurit Nirel**

Israel

**Charles Normand**

Ireland

**Ora Paltiel**

Israel

**Elyse Park**

United States of America

**L Gregory Pawlson**

United States of America

**Joseph Pliskin**

Israel

**Avi Porath**

Israel

**Nosaiba Rayan**

Israel

**Brian Reichman**

Israel

**Shmuel Rishpon**

Israel

**David Roe**

Israel

**Alvin Roth**

United States of America

**Siegal Sadetzki**

Israel

**Richard Saltman**

United States of America

**Jörg Schaaber**

Germany

**Stephen Schoenbaum**

United States of America

**Michal Schuster**

Israel

**Efrat Shadmi**

Israel

**Sigal Shafran -Tikva**

Israel

**Carmel Shalev**

Israel

**Varda Shalev**

Israel

**Segev Shani**

Israel

**Nathan Shapiro**

United States of America

**Yaniv Sherer**

Israel

**Amir Shmueli**

Israel

**Judith Shuval**

Israel

**Pierre Singer**

Israel

**Shepherd Roee Singer**

Israel

**Varda Soskolne**

Israel

**Ruth Stalnikowicz**

Israel

**Avraham Steinberg**

Israel

**Tzipi Strauss**

Israel

**Orly Toren**

Israel

**Lori Uscher-Pines**

United States of America

**Wynand van de Ven**

Netherlands

**Baruch Velan**

Israel

**Shlomo Vinker**

Israel

**Charles Weissman**

Israel

**Rachel Wilf Miron**

Israel

**Raphael Wittenberg**

United Kingdom

**Pnina Zadka**

Israel

